# The Bacterial Nanorecorder: Engineering *E. coli* to Function as a Chemical Recording Device

**DOI:** 10.1371/journal.pone.0027559

**Published:** 2011-11-23

**Authors:** Prasanna Bhomkar, Wayne Materi, David S. Wishart

**Affiliations:** 1 National Institute for Nanotechnology, Edmonton, Alberta, Canada; 2 Department of Computing Science, University of Alberta, Edmonton, Alberta, Canada; 3 Department of Biological Sciences, University of Alberta, Edmonton, Alberta, Canada; Center for Genomic Regulation, Spain

## Abstract

Synthetic biology is an emerging branch of molecular biology that uses synthetic genetic constructs to create man-made cells or organisms that are capable of performing novel and/or useful applications. Using a synthetic chemically sensitive genetic toggle switch to activate appropriate fluorescent protein indicators (GFP, RFP) and a cell division inhibitor (minC), we have created a novel *E. coli* strain that can be used as a highly specific, yet simple and inexpensive chemical recording device. This biological “nanorecorder” can be used to determine both the type and the time at which a brief chemical exposure event has occurred. In particular, we show that the short–term exposure (15–30 min) of cells harboring this synthetic genetic circuit to small molecule signals (anhydrotetracycline or IPTG) triggered long-term and uniform cell elongation, with cell length being directly proportional to the time elapsed following a brief chemical exposure. This work demonstrates that facile modification of an existing genetic toggle switch can be exploited to generate a robust, biologically-based “nanorecorder” that could potentially be adapted to detect, respond and record a wide range of chemical stimuli that may vary over time and space.

## Introduction

Synthetic biology or “synbio” is an emerging field of biotechnology that combines molecular biology with genetic engineering and protein engineering. Like synthetic chemistry, synthetic biology involves the design and synthesis of novel entities with unusual and/or useful functionalities. In synthetic biology, the raw materials are DNA and proteins, while in synthetic chemistry the raw materials are small molecule chemicals and chemical catalysts. Over the past decade, synthetic biologists have designed and developed a number of “synthetic prokaryotes” exhibiting completely novel or entirely un-natural functions such as a bacterial oscillator [1], a toggle switch [2], a bacterial photocopier [3], a bacterial drug delivery system [4], a biofilm-destroying agent [5], an edge detection tool [6] and a bacterial clock [7]. More recently the same synbio concepts have been successfully applied to eukaryotic systems [8]–[11]. However, constructing synthetic cells or organisms that respond in a robust and precise manner still represents a significant challenge for today's synthetic biologists. In particular, gaps in our understanding of the operational principles of natural biological systems, coupled with the lack of tools to fully characterize synbio-inspired devices has led to many more proof-of-principle demonstrations than useful applications [12].

The bistable toggle switch is an example of a genetic “memory element” that is among the most robust and useful synthetic biology devices developed to date. This device is a synthetic genetic circuit that generates a sustained cellular response to a transient stimulus [2]. In its initial inception, the toggle switch consisted of two constitutive promoters driving the expression of mutually inhibitory repressor proteins. Other genetic memory element circuits include the *fimE*-driven inversion recombination switch [13], the conditional memory circuit, wherein a bistable toggle switch is modified by the addition of two regulatory proteins [14] and the riboregulator-/recombinase-based genetic counter [15]. Because of its reliance on relatively few regulatory elements, the toggle switch design is relatively tolerant of stochastic fluctuations and has been exploited in a number of synbio applications. These include an *E.coli* strain that responds by biofilm formation upon transient exposure to a DNA-damaging agent [16] and a cadmium biosensing *Pseudomonas putida* strain [17].

Although the toggle switch represents an effective way to detect and store the “memory” of an event, it is currently not possible to “time stamp” such a memory using the current toggle switch configuration. In other words, it is not yet feasible to determine the time when the toggle switch was initially exposed to a stimulus or signal molecule without a continuous recording of the reporter gene expression. Time stamping would obviously be a useful feature for biologically based chemosensors, particularly if one is interested in passive environmental monitoring, tracking the occurrences of pulsed chemical releases or performing longer term longitudinal chemosensing. This time stamp problem arises because most synbio systems employing toggle switches have relied on the expression of primary output responses that do not increase predictably or in a retroactively measurable fashion following exposure to the signal molecule. Indeed most biosensor or chemosensor output responses are typically limited to fluorescent reporter proteins (namely GFP) or phenotypic alterations (viz. biofilm formation) which cannot be readily used as a quantitative measure of the time elapsed following an initial (or a brief) signal exposure. Also, the visualization of a fluorescent reporter protein necessitates the use of a well-equipped microscope facility and hence cannot be used for routine or field-based detection.

To address this time-stamping and detection limitation with today's bacterial biosensors we decided to utilize a number of pre-existing synbio circuits to create a time-stamping, chemo-sensing bacterium or more simply, a bacterial “nanorecorder”. This bacterial time-recording device has been designed so that it could 1) respond to a brief event or stimulus; 2) maintain a memory of that event/stimulus; and 3) time stamp that event or stimulus. In other words we wanted to create a synthetic bacterium that could register a long-term phenotypic response following short-term exposure to a chemical and utilize the elicited phenotypic response to estimate the time elapsed after chemical exposure. One suitable phenotypic response capable of being able to time-stamp events in bacteria could be to arrest the cell division process, and instead activate cell elongation. Utilizing this response has three benefits: 1) the detection of activated cells becomes much easier (light microsopy vs. fluorescence microscopy); 2) the length of the cell is proportional to the time following exposure and 3) the system is self-limiting (cells stop generating viable progeny after exposure to the chemical stimulus). Therefore to create this bacterial time-recording device we coupled the expression of the cell division inhibitor gene, *minC* [18]–[20], with a standard genetic toggle switch to engineer a phenotypic response (i.e. filamentation) that can be used to calculate the time of initial signal exposure. Specifically, in this report we describe the modification of two well-studied bistable toggle plasmids (namely, pTAK117 and pIKE107) [2] to support the co-expression of physiologically relevant levels of MinC protein. Transforming these plasmids into suitable host cells led to the production of *E. coli* strains that fluoresce with different colors (depending on the type of chemical exposure) and elongate up to 30× their normal length (allowing quantitative determination of time elapsed following initial signal exposure) upon short-term exposure to different signal molecules (namely IPTG or anhydrotetracycline). The length of the cells corresponds to the time (in cell division “units”) since exposure or activation, allowing quantitative determination of time elapsed following initial signal exposure. By analogy to the manner in which the length of paper produced from a chart recorder corresponds to the length of time since the start of recording, this system resembles a recorder device. The term “nanorecorder” was coined because this system uses specially engineered nanoscale devices, including proteins, plasmids and bacteria (width = 500 nm), to create a functioning recorder.

This work demonstrates how the careful modification of an existing genetic toggle switch can be exploited to generate a biologically-based “nanorecorder” that could potentially be adapted to detect, respond, recall and record a wide range of chemical stimuli that may vary over time and space.

## Materials and Methods

### Bacterial strains, growth conditions and chemicals

*E. coli* strain XL-1-Blue (obtained from Stratagene, USA) was used for the construction of all plasmids described in this work. The *E. coli* strains PB103 [21], PB103:pDR175 and PB103:λ DR144 were obtained as gifts from Dr. Piet A. J. de Boer (Case Western Reserve University, Cleveland, OH). Plasmid pDR175 [20] is a pGB2 [22] derivative wherein expression of the MinC protein is induced by a 42°C heat-shock, while the integrated prophage λ DR144 (*imm* ^21^ *bla^+^ lacI* ^q+^ P*_lac_*::*sfiA*) encodes an IPTG-inducible SfiA protein. The host strain PB103 was used to test the effect of expression of two different cell division inhibitors, namely MinC and SfiA, on cell elongation. Cells were grown in Luria-Bertani (LB) medium at 37°C and plasmids were selected using 50 mg/L ampicillin and 25 mg/L spectinomycin. Ampicillin, spectinomycin and IPTG were procured from Gold Biotechnology, MO, Anhydrotetracycline was manufactured using our in-house chemical synthesis facility, while LB medium was prepared using Difco™ LB broth Miller (BD Biosciences, Canada). Restriction enzymes were obtained from New England Biolabs, Canada and primer sequences were commercially synthesized from Integrated DNA Technologies, Canada. For expression studies, the cells were cultured at 31°C. All molecular cloning manipulations were performed according to standard protocols described in the Molecular Cloning Manual [23].

### Plasmid construction

**Figure 1:**
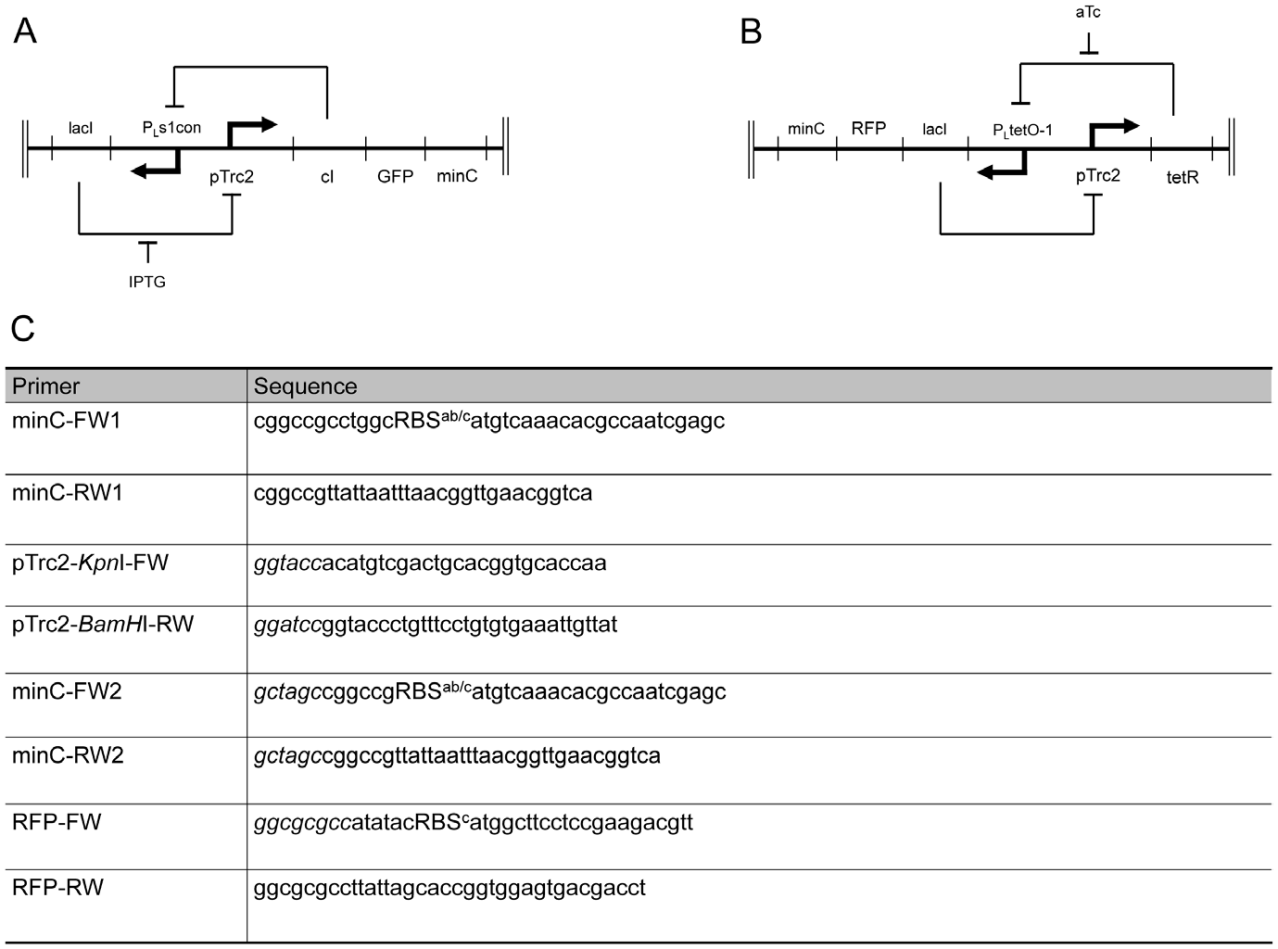
Representation of modified toggle switch plasmids and primer sequences. A. pTAK117-minC functions as an IPTG-sensitive nanorecorder. The repressor, cI, inhibits transcription from the PLs1con promoter while the lacI repressor inhibits transcription from the pTrc2 promoter. Transient addition of IPTG derepresses pTrc2 and initiates long-term expression of GFP and MinC reporter genes. B. pIKE107-RFP-minC is the anhydrotetracycline-responsive nanorecorder. The lacI repressor prevents transcription from the pTrc2 promoter while the TetR repressor inhibits transcption initiation from the PLtetO-1 promoter. Transient exposure to anhydrotetracycline relieves PLtetO-1 from TetR-mediated repression, stimulating sustained expression of RFP and minC reporter genes. C. Primers used for construction of toggle switch plasmids. RBS represents ribosome binding sequences, RBS^a^: aaagaggagaaa, RBS^b^: tcacacaggaaag, RBS^c^: tcacacaggaaacc. The sequences in italics represent the restriction sites used for cloning.

Toggle switch plasmids, pTAK117 and pIKE107 [2], were obtained as gifts from Dr. Jim Collins (Centre for Biodynamics, Boston, MA) and used as the basis for constructing derivative plasmids. The pTAK117 derivative, pTAK117-minC, was designed to express green fluorescent protein (GFP) and MinC upon exposure to IPTG, while the pIKE107 derivative, pIKE107-RFP-minC, was designed to express red fluorescent protein (RFP) and MinC upon exposure to anhydrotetracycline (i.e. aTc). The *minC* coding sequence was amplified by PCR using *E. coli* genomic DNA (strain MG1655, ATCC number 700926D-5) as a template with the primers minC-FW1 and minC-RW1 and ligated into PCR 2.1-TOPO (Invitrogen, Canada) to obtain PCR 2.1-minC. The 5′ primer sequence also included ribosome-binding sites (RBSs) of different strengths to confer translational control on MinC expression levels. The IPTG-inducible promoter, pTrc2, was amplified using pTAK117 as a template with primers (pTrc2-*Kpn*I-FW, pTrc2-*BamH*I-RW) and cloned into pGEMT. The pTrc2 promoter fragment (*Kpn*I-*BamH*I) was ligated into *Kpn*I-*BamH*I digested PCR 2.1-minC to obtain pTrc-minC. This plasmid was co-transformed with the toggle switch plasmid, pTAK117, into the PB103 host cells by heat shock transformation and co-transformants were selected on ampicillin and kanamycin. In independent experiments, the *minC* coding sequence was amplified using primers having *Nhe*I overhangs at both ends (minC-FW2, minC-RW2) and cloned downstream of the GFP gene into *Nhe*I-digested pTAK117. The ligation mixture was transformed into XL-1 blue host cells and plasmid DNA was isolated from all the putative recombinant clones (regardless of the *minC* orientation), followed by independently transforming the isolated plasmids into PB103 host cells. Recombinants having *minC* gene cloned in the sense orientation (with respect to the pTrc2 promoter) were identified using a phenotypic screen (as described in the Results and Discussion section), confirmed by plasmid DNA sequencing and were designated as pTAK117-minC (Figure 1A). A “toggle-less” version of the IPTG-nanorecorder (pTAK117-minC*) was made by digesting pTAK117-minC with *Sph*I-*Kpn*I (to get rid of the pTrc2-cI fragment) followed by ligating a commercially synthesized *Sph*I-*Kpn*I fragment (comprising the pTrc2 promoter). Due to this manipulation, PB103 cells transformed pTAK117-minC* would be unable to synthesize the cI repressor and hence require continuous IPTG exposure to induce GFP and MinC expression.

In order to construct pIKE107-RFP-minC, GFP was excised from pIKE107 by a *Kpn*I-*Nhe*I digest followed by blunting and self-ligation of the vector backbone. The RFP gene was then amplified from the Biobrick plasmid BBa_J23018 using primers having *Nhe*I overhangs at both ends (RFP-FW, RFP-RW), subcloned into PCR2.1-TOPO (to obtain PCR2.1-TOPO-RFP) before its ligation as an *Asc*I fragment in *Asc*I-digested pIKE107 to obtain pIKE107-RFP. The RFP-FW primer sequence included the RBS^c^ (used earlier to control MinC expression in pTAK117). The *minC* fragment (obtained as an *Nhe*I digest from pTAK117-minC) was blunt-ended using T4 DNA polymerase and ligated (downstream of RFP) in *Nhe*I-digested and blunt-ended pIKE107-RFP. Recombinant clones wherein *minC* was ligated in the sense orientation (with respect to the anhydrotetracycline-inducible P_L_tetO-1 promoter) were identified using the phenotypic screen, validated using DNA sequencing and were designated as pIKE107-RFP-minC (Figure 1B). Sequences for the primers used in these experiments are listed in Figure 1C. Restriction maps of pTAK117-minC, pIKE107-RFP-minC and the nucleotide sequence of the commercially synthesized *Sph*I-*Kpn*I fragment are provided in Supporting information as Figures S1 and S2 respectively.

### Characterization of the bacterial nanorecorders

The IPTG-inducible version of the bacterial nanorecorder strain (PB103:pTAK117-minC) was characterized in order to determine the limits of its sensitivity and its potential suitability as a time-stamping or recording device. To monitor the behavior of the cell population under continuous IPTG induction, the cells were grown in the presence of 2 mM IPTG at 31°C and aliquots were observed via microscopy at 30 min-intervals to record their fluorescence signal intensity and cell length. In order to determine the minimum IPTG concentration and the shortest time of cellular exposure necessary to register a long-term response, the cells were incubated with 2 mM, 5 mM, 10 mM and 20 mM IPTG for 15 min, 30 min and 60 min. After exposure the cells were briefly pelleted, washed twice in LB medium before suspending them in LB (50 mg/L ampicillin, without IPTG) and retransferred to culture tubes for further growth. The cells were observed at 30-min intervals to record their fluorescence signal intensity and cell length. Aliquots from the same culture grown with 2 mM IPTG and in IPTG-less media were included as positive and negative controls. The anhydrotetracycline-inducible version of the bacterial nanorecorder strain (PB103:pIKE107-RFP-minC) was characterized similarly upon exposure to 0.05 nM, 0.1 nM, 0.25 nM, 0.5 nM, 1 nM, 2 nM, 5 nM and 10 nM anhydrotetracycline and the responses obtained were compared to those obtained with the IPTG-inducible version of the nanorecorder.

### Microscopy, data collection and analysis

**Figure 2:**
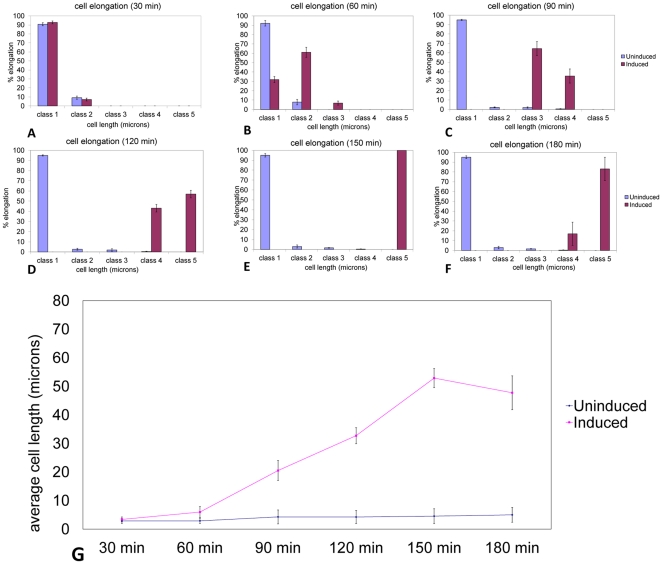
Time course of cell elongation upon continuous exposure to 2 mM IPTG. PB103:pTAK117-minC cells observed at A) 30 min, B) 60 min, C) 90 min, D) 120 min, E) 150 min and F) 180 min post-IPTG exposure. Class sizes are as follows: class 1: 0.5 µm–4 µm, class 2: 4.1 µm–8 µm, class 3: 8.1 µm–16 µm, class 4: 16.1 µm–32 µm, class 5: 32.1 µm–64 µm, class 6: 64.1 µm–132 µm. G. *Average cell length versus elapsed time upon constant exposure to 2 mM IPTG*. PB103:pTAK117-minC cells observed at different time intervals (30 min, 60 min, 90 min, 120 min, 150 min and 180 min) following continuous IPTG exposure to 2 mM IPTG following which the averaged cell length was plotted with respect to time elapsed post-IPTG exposure.

Twenty microlitres of actively growing cultures were transferred to a glass slide and visualized by phase contrast microscopy at 20× magnification (Olympus IX81 inverted microscope). Images were collected using Image Pro Plus software (Media Cybernetics). To measure cell length, the image was calibrated to the magnification used and cells selected using the software's “Select ranges” function. The selected cells were distributed into various bin sizes (class 1: 0.5 µm–4 µm, class 2: 4.1 µm–8 µm, class 3: 8.1 µm–16 µm, class 4: 16.1 µm–32 µm, class 5: 32.1 µm–64 µm, class 6: 64.1 µm–132 µm) using the “Count” and “Measure” functions of the software. Cells in class 1 correspond to those having gone through one doubling without cytokinesis; cells in class 2 correspond to those having gone through two doublings without cytokinesis; cells in class 3 correspond to those that have doubled at least three times without cytokinesis, cells in class 4 correspond to those that have doubled at least four times without cytokinesis and cells in class 5 correspond to those that have doubled five times or more without cytokinesis. Note that a range of cell lengths are used for each doubling category because the variable orientation of the cells in the microscope viewing plane causes optical foreshortening of the true cell length. The data obtained was transferred to MS Excel and used to calculate the number of cells measured in each class (Figure 2 A–F) and the corresponding standard deviation of the cell length distribution for each class. All measurements were obtained from three independent cultures maintained under identical conditions. Each experiment was repeated at least three times. For each treatment, at least 500 cells were counted. The average cell length was also plotted versus time elapsed for better data visualization (Figure 2G). GFP and RFP expression in cells was visualized under UV fluorescence using FITC and TRITC filters, respectively.

## Results and Discussion

Synthetic biology relies on the construction of man-made or synthetic genetic devices to produce cells with predictable yet fundamentally un-natural properties. This is most often done by harnessing and modifying endogenous pathways to generate artificial genetic networks. For example, the genetic bistable toggle switch [2] has been exploited as a means to program cells using a modular design strategy, thereby paving the way for the development of “plug-and-play” genetic circuit devices. A number of synthetic gene networks designed to function as memory elements have already been described [13]–[16]. However, these systems cannot be used to estimate the time elapsed following exposure (either brief or continuous) to a signal molecule. Because of this limitation we decided to generate a bacterial time-stamp device (a biological nanorecorder) that, upon transient (or continuous) exposure to a signal molecule, would generate an easily quantifiable phenotypic response that would allow us to estimate the time elapsed following signal exposure.

**Figure 3:**
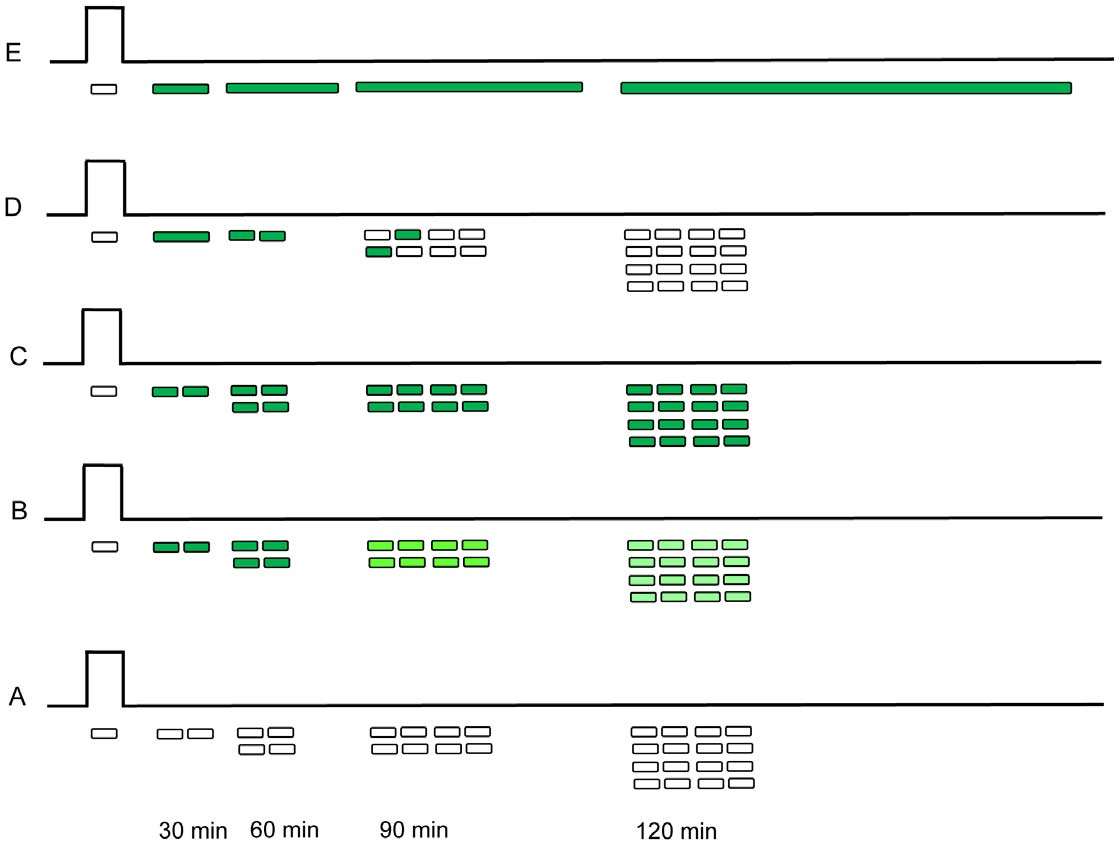
Schematic illustration of the proposed bacterial time-stamp devices. Panel B outlines the expected behavior of cells wherein transient exposure to a signal molecule rapidly induces expression of a fluorescent reporter protein followed by periodic reduction in fluorescent intensity upon removal of the signal molecule. Brief signal exposure of bacteria containing only the toggle switch driving expression of a fluorescent reporter protein (C) would result in fluorescence in the dividing progeny, while identical signal exposure in bacteria containing a plasmid capable of constitutive expression of a cell-division inhibitor and a fluorescent reporter protein (D) would eventually result in non-elongating and non-fluorescent progeny. Only bacteria containing a toggle switch driving expression of a fluorescent reporter protein and a cell-division inhibitor protein (E) are expected to display fluorescence and sustained cellular elongation in progenitor cells, the length of which could be used to calculate time elapsed following signal exposure. Control bacteria devoid of toggle switch are represented as (A).

Figure 3 conceptualizes the expected behavior of the proposed time stamp devices. It describes what we expect to happen if we prepared a bacterial system that contained i) only a chemically activated toggle switch coupled to a fluorescent signal, ii) no toggle switch but had chemically activated cell elongation coupled to a fluorescent signal, and iii) both a chemically activated toggle switch and a cell division inhibitor coupled to a fluorescent signal. As part of the proposed time stamp devices, we first explored the feasibility of an alternative approach for time recording, wherein transient exposure to a signal molecule rapidly induces expression of a fluorescent reporter and the time elapsed following signal exposure can be estimated by monitoring the periodic reduction in fluorescence intensity (Figure 3B). We found that this approach could be used to time-stamp a 30 min pulse for 3 h and a 45 min pulse for 4 h (see Supporting information, Figure S3 for details). However, we experienced that measuring fluorescence intensity and quantifying fluorescent decay was relatively tedious and time consuming (as one needs to carefully normalize fluorescence intensities) and is very sensitive to minor changes in the focal plane and could be easily affected external parameters (such as the life of the mercury lamp used for visualizing fluorescence). Additionally, fluorescence intensities were also dependent on media used for cell culture (intensities were higher by up to 20% in M9 medium as compared to LB medium). Although there could be promoter-reporter-strain-media combinations where the fluorescence-intensity based method could yield better results, we explored a toggle-switch based approach to time-stamping. Accordingly, we were interested to investigate cell length as an alternative reporter system because cell length can be easily visualized using a simple light microscope, can be potentially quantified without the use of sophisticated software and is independent of media type. As shown in Figure 3C, exposing bacteria containing only the toggle switch-fluorescent reporter combination to a short pulse of the signaling molecule would result in many fluorescent progeny but no elongated cells. While the presence of fluorescent cells would indicate an exposure event, without knowing the initial number of cells that were exposed or the number of cells that were activated, it would not be possible to calculate when the exposure event occurred. As shown in Figure 3D, exposing bacteria containing a combination of a fluorescent reporter and cell division inhibitor but no toggle switch, to a short pulse of the signaling molecule would temporarily inhibit division in the progenitor cells but after a short time the cells would lose their memory of the exposure event and essentially nothing useful could be detected. Finally, as shown in Figure 3E, exposing bacteria containing both the toggle switch and the cell division inhibitor to the signaling molecule would result in an easily detected phenotypic response where the length of the elongating bacterial cell could be used to calculate the time elapsed following exposure. Based on these scenarios, we constructed a bacterial system that contained a chemically activated toggle switch and a cell division inhibitor coupled to a fluorescent signal. Balancing physiological levels of this reporter gene (*minC*) in bacterial cells proved to be critical to the development of this kind of bacterial nanorecorder (For further details, refer to Supporting information, Text S1-Optimization of the MinC reporter system. For example, Figure S4 demonstrates that MinC expression resulted in better inhibition of cell division than SfiA expression).

### Characterization of the IPTG-sensitive bacterial “time-stamp” device

The following experiments were initiated to explore the potential suitability of PB103 cells harboring the modified IPTG toggle switch (pTAK117-minC) as a “time-stamp” device and determine the limits of its sensitivity.

#### Time course of cell elongation upon continuous IPTG exposure

PB103:pTAK117 cells cultured under continuous exposure to 2 mM IPTG at 31°C in LB medium were 2 µm long and divided every 35 min in their exponential phase. On the other hand, PB103 cells (transformed with the modified toggle switch, pTAK117-minC) cultured under identical conditions displayed uniform cell elongation in a time-dependent manner (Figure 2). After 30 min of incubation, the PB103:pTAK117-minC cell population showed no visible signs of cell elongation in either the induced or non-induced treatments (Figure 2). However, after 60 min of incubation (Figure 2B), the induced cell population comprised of 61% cells in class 2 (4.1 µm–8 µm) and 8% cells in class 3 (8.1 µm–16 µm). The induced cell population continued to elongate in a time-dependent manner and reached maximal cell lengths after 150 min (Figure 2E), wherein all the cells were found to be in class 5 (32.1 µm–64 µm). Further cell elongation was not observed beyond this incubation period. Meanwhile, after 180 min of incubation more than 90% of the uninduced PB103:pTAK117-minC cell population consisted of cells in class 1 (0.5 µm–4 µm), thereby displaying negligible background elongation in the absence of IPTG.

The maximal length of the filaments obtained upon continuous exposure of the PB103:pTAK117-minC to 2 mM IPTG was 58 µm (nearly 30× the length of their uninduced counterparts). Arends and Weiss [24] noted that long-term inhibition of cell division in *E.coli* apparently did not affect the growth rate, DNA replication or chromosome segregation and the only obvious effects were morphological wherein these cells grew into long, aseptate filaments that ultimately lysed, eventually resulting in lethality.

Overall, we found that cell elongation of PB103 cells (pTAK117-minC), following exposure to the signal molecule (IPTG), occurred in a time-dependent manner and that it was accompanied by the appearance of different-sized cells at discrete time intervals up to 2.5 h. These data suggest that cell length can be used to detect and calculate the time elapsed following exposure to a signal molecule. To illustrate, a cell population consisting of ca. 60% cells in class 3 (8.1 µm–16 µm) and 40% cells in class 4 (16.1 µm–32 µm) would suggest that these cells were exposed to the signal molecule 90 min (3–4 doublings) prior to measurement, while a cell population consisting of ca. 40% cells in class 4 (16.1 µm–32 µm) and 60% cells in class 5 (32.1 µm–64 µm) would be indicative of an exposure event that occurred 120 min (4–5 doublings) in the past. It should be noted that because cell doubling times are dependent on growth medium and culture temperature, we would advocate that the nanorecorder's time should ideally be measured in units of cell doublings rather than in standard units of hours or minutes. Plotting the average cell length (without binning the cells into different class sizes) versus elapsed time also demonstrated that there was a consistent increase in the average measured cell length for up to 150 min of continuous IPTG exposure (Figure 2G).

#### Utility and sensitivity of cell elongation and toggle-switching as a time-stamp device

Upon confirming that continuous signal molecule (IPTG) exposure of PB103 containing the modified toggle switch plasmid (pTAK117-minC) led to the phenotype of interest, (i.e. chemo-selective time-dependent cell elongation), we decided to determine the utility and sensitivity limits of this IPTG nanorecorder. In particular, we wanted to answer four questions: 1) What is the minimum length of time and minimum IPTG concentration needed to activate the nanorecorder? Within the context of the MinC reporter system 2) Is the toggle switch necessary for proper time stamping? 3) Is continuous exposure of a chemical stimulus required to activate cells without the toggle switch? and 4) Is cell elongation absolutely necessary to record time stamping or can it be inferred by other means? To answer these questions we prepared two separate *E. coli* strains. The first strain consisted of PB103 cells transformed with a “toggle-less” version of pTAK117-minC (i.e. PB103:pTAK117-minC*) that is incapable of synthesizing the cI repressor and hence express MinC only upon continuous IPTG exposure. The second strain consisted of PB103 cells transformed with the original toggle switch plasmid (pTAK117). These PB103:pTAK117 cells do not express MinC and therefore continue to divide normally following exposure to IPTG.

**Figure 4:**
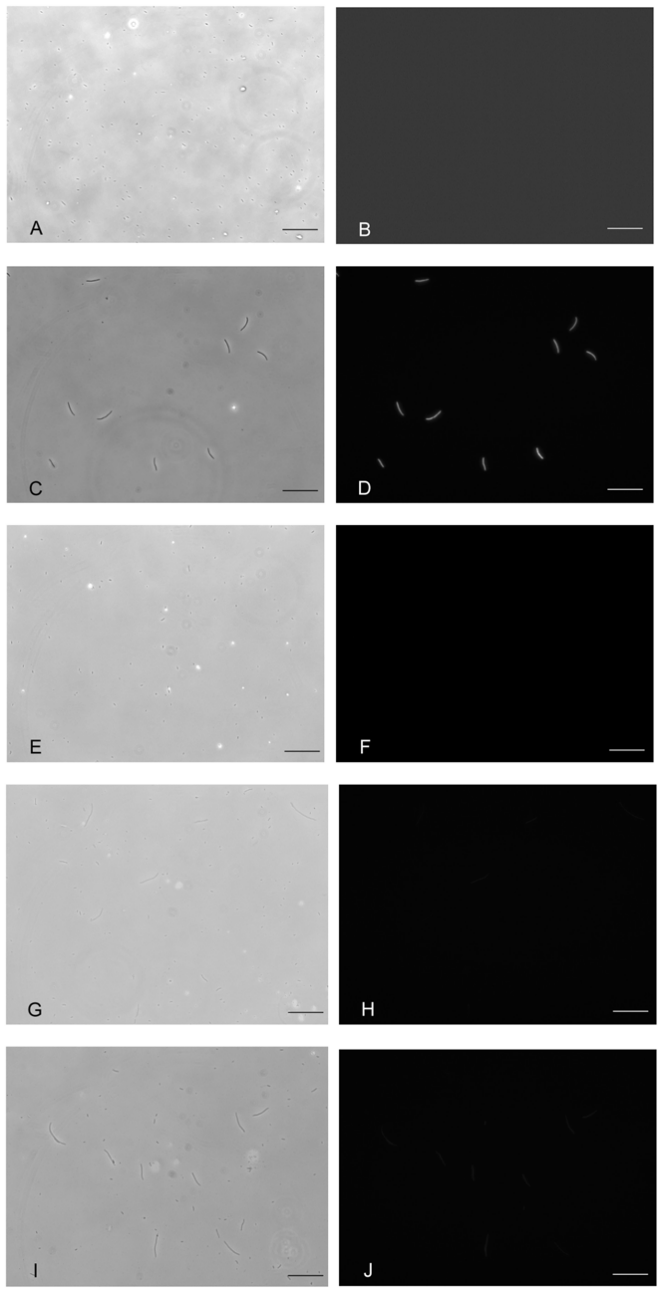
“Memory effect” upon short-term IPTG exposure. Visible light and UV-light images of PB103: p TAK117-minC exposed to 10 mM IPTG for 15 min (E, F),30 min (G, H) and 60 min (I, J) before washing and resuspending the cells in IPTG-less LB amp50 medium. Controls include cells not exposed to IPTG (A, B) and cells grown under continuous exposure to 2 mM IPTG (C, D). Images were taken after 2 h 30 min of exposure to 10 mM IPTG. Scale bar is 50 µm.

Gardner et al. [2] reported that the toggle switch plasmid, pTAK117, begins switching from the “low” state (pTrc2 repressed, no cI repressor, no GFP) to the “high” state (i.e. derepression of pTrc2 leading to cI and GFP expression) after 3–4 h of IPTG induction. To address the first question, PB103 cells transformed with pTAK117-minC were exposed to 2 mM, 5 mM, 10 mM and 20 mM IPTG for 15 min, 30 min and 60 min. After exposure, the 12 sets of cells were pelleted and washed twice with plain LB medium before their final resuspension and incubation in IPTG-less LB amp50 medium. The exposed cultures were monitored for 180 min post-IPTG exposure and the population was sorted into different classes based on observed cell lengths. Irrespective of the duration of exposure, PB103:pTAK117-minC cells exposed to 2 mM or 5 mM IPTG did not elongate or express GFP after transfer to IPTG-less growth medium. Filamentation was observed in cultures exposed to 10 mM (and 20 mM) IPTG for 30 min (Figure 4G). In other words, the IPTG nanorecorder required at least a 30 min exposure to a minimum concentration of 10 mM IPTG to register the presence of the chemical signal and begin recording. After 180 min, 62% of the induced nanorecorder cell population belonged to class 1 (0.5 µm–4 µm), 6% was in class 2 (4.1 µm–8 µm) and 32% of the population belonged to class 3 (8.1 µm–16 µm). Instead of calculating the *averaged* length of all the transiently cells induced cells, we could estimate the time elapsed post-signal exposure by measuring length of the most elongated cells. Also, GFP expression was observed in all filamenting cells exposed to a 30 min, 10 mM IPTG pulse, but the GFP intensity was 3–4 fold lower than seen in PB103:pTAK117-minC cells that were grown under the continuous presence of 2 mM IPTG (Figure 4H). Further increases in the frequency (i.e. total number of filamentous cells) or the extent of cell filamentation (i.e. the increase in filament lengths of the responding cell population) were not observed upon increasing the duration of cell exposure to 60 min (at 10 mM IPTG) or upon increasing the IPTG concentration (e.g. to 20 mM). Therefore, our results indicate that the switching process is dependent on the concentration of the signal molecule and that the bacterial nanorecorder can begin switching from the “low” to “high” state at 30 min post-exposure to 10 mM IPTG.

By contrast, the PB103:pTAK117-minC* cells (the “toggle-less” version) could filament and express GFP only upon continuous exposure to 2 mM IPTG. Therefore these toggle-less cells had no “memory” of a short-term exposure event, although they could effectively record the duration of continuous signal exposure. In parallel experiments performed with PB103:pTAK117, we found that 56% of the population expressed GFP when exposed to 10 mM IPTG for 30 min. As expected, none of these cells showed any elongation. It is worth noting that with these PB103:pTAK117 cells, the level of fluorescence decayed after 24 h post-transfer to IPTG-less growth medium. Therefore these continuously dividing cells had only a partial or fading “memory” of an exposure event. Consequently they could not be used to effectively record the duration or the time since IPTG exposure. The results of these control experiments show that the use of a toggle switch in combination with cell elongation and an optional chemo-specific fluorescent signal is an effective route to generating an easy-to-visualize and hence useable nanorecorder. It is also worth mentioning that PB103:pTAK117 cells could express GFP for up to 48 h post-IPTG exposure, but this was only possible if the cells were subcultured at 3-h intervals into fresh growth media.

Our results with the pTAK117 plasmid (Supporting information, Figure S5) were interesting, especially in light of what is currently understood about toggle switch behavior. As noted earlier, Gardner et al. [2] reported that the toggle switch plasmid began switching from the “low” (no GFP expression) to the “high” (i.e. GFP expressing) state after three hours of exposure to 2 mM IPTG. We observed that PB103:pTAK117 cells exposed for as little as 30 min expressed GFP (Figure S5-D), although a higher concentration of IPTG (10 mM) was required. This suggests that exposing cells to higher IPTG concentrations results in faster quenching of the unbound intracellular LacI repressor molecules as well as quickly derepressing the pTrc2-bound LacI repressor, thereby reducing the exposure time necessary to toggle the genetic switch from a “low” to a “high” state.

Although phenotypic diversity of a clonal bacterial culture is stochastic [25], such cells also display subpopulations with heritable phenotypic differences [26]. The toggle switch is a cyclic digenic (two-gene) system comprised of two promoter-repressor cassettes, arranged so that each promoter is inhibited by the repressor expressed by the opposing promoter. This framework is considered robust enough to tolerate inherent stochastic fluctuations in gene expression. Consequently it will switch to either of the two alternate stable states only by the addition of specific external inducers (such as IPTG and temperature in the case of the toggle plasmid pTAK117). Our demonstration that the “memory response”, characteristic of the toggle switch, is obtained only when cells are kept at low density (through continuous subculturing every 3 h) corroborates the findings of Stupak et al. [27] that the functional state of a bistable toggle switch in the absence of inducers is also dependent on bacterial cell metabolism. In their study, Stupak et al. [27] demonstrated that increasing the time interval between cell subculture lowered the cellular growth rate, decreased the level of metabolism and resulted in the switch of these cyclic systems from bistable to monostable functioning regime. While decreased levels of cellular metabolism are known to result in stochastic changes in the ratios of cellular proteins, Stupak et al. [27] also proposed that a low cellular growth rate could also affect the levels and/or stability of the repressor proteins that regulate the toggle switch. Although other mechanisms, which may alter the levels of the repressor proteins or other components of the toggle switch, cannot be ruled out, our study suggests that stochastic fluctuations in gene expression may be accentuated by subtle changes in growth conditions and result in long-term changes in epigenotypes. In the reverse case scenario, Tan et al. [28] reported that changes in bacterial host physiology were responsible for a counter-intuitive situation, wherein bistable gene expression was displayed by a simple positive feedback circuit. These studies along with our results suggest that unexpected interactions between a genetic circuit and its host can result in unintended perturbations in the dynamics of gene expression. Therefore, properly accounting for these factors is important for engineering any kind of robust behavior in synthetic biological systems.

### Anhydrotetracycline-sensitive version of the bacterial “nanocrecorder”

Similar in configuration to pTAK117, the anhydrotetracycline-sensitive toggle plasmid, pIKE107, switches from the “high” state (reporter gene expression) to the “low” state (no expression of reporter genes) upon short-term exposure to anhydrotetracycline. Using this toggle switch we decided to make a anhydrotetracycline-responsive version of the “time-stamp” device that would express the reporter genes upon anhydrotetracycline exposure. While MinC expression remained our choice for phenotype-based detection of an exposure event, we chose to express the red fluorescent protein (RFP) to differentiate its read-out response from the GFP-based output obtained from the IPTG-nanorecorder. This modification enabled colour-coded chemo-selective detection.

#### Optimizing expression of RFP reporter gene

Although RFP expression was detected both in *E. coli* transformed with a template Biobrick plasmid (i.e. BBa_J23018) and in *E. coli* transformed with PCR2.1-TOPO-RFP (data not shown), our initial attempts to express the RFP coding sequence in pIKE107 were unsuccessful. Pfleger et al. [29] demonstrated that the problems related to RFP expression in *E. coli* were due to the formation of strong base-pairing between a GC-rich region present near the 5′ end of the RFP gene and the RBS used for translational initiation. Using saturation mutagenesis, Pfleger et al. [29] modified the nucleotides of these 5′ RFP codons (without altering the amino acid sequences) and effectively restored RFP expression by minimizing base-pairing between the 5′ end of the RFP gene and the RBS. Similarly, we hypothesized that our inability to obtain expression of the RFP-encoding open reading frame upon transfer from a Biobrick plasmid (BBa_J23018) to a toggle plasmid (pIKE107) stemmed from the potential base-pairing between the 5′ RFP sequence and the RBS used for translational initiation. Based on this premise, the RFP gene amplified using the native Biobrick RBS (identified by sequencing BBa_J23018) was able to express RFP when cloned into pIKE107. A comparison of the RBS's used for RFP expression using a secondary structure prediction program did not suggest any differences in their base pairing abilities with the 5′ end of the RFP gene. It is therefore possible that the Biobrick RBS was stronger and was responsible for RFP expression in pIKE107.

#### Comparative behavior of the IPTG- and aTc-sensitive time stamp devices

**Figure 5:**
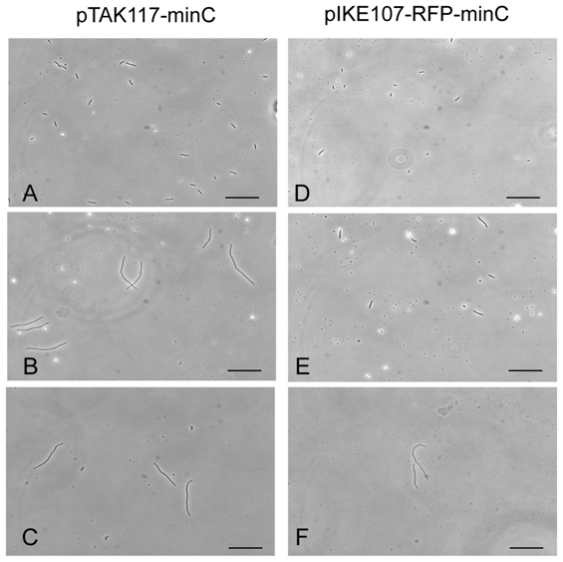
Comparative behavior of the IPTG- and aTc-sensitive time stamp devices. Visible light images of PB103:pTAK117-minC and PB103:pIKE107-RFP-minC upon continuous exposure to 2 mM IPTG and 1 nM anhydrotetracycline, respectively. Overnight cultures (initiated from glycerol stocks and grown at 37°C in LB amp50) were sub-cultured to fresh LB amp50 for 90 min and used at a 100-fold dilution to inoculate fresh LB amp50 supplemented with IPTG and anhydrotetracycline. Cells were observed after 90 min (A, D); 2 h 30 min (B, E) and 4 h (C, F). Scale bar is 50 µm.

**Figure 6:**
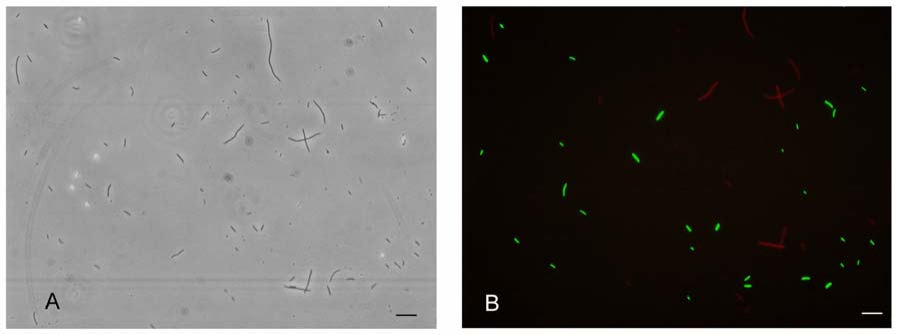
Response of mixed culture IPTG- and aTc-sensitive nanorecorders. Visible light (A) and false coloured fluorescence microscopy image of a mixed culture of PB103:pTAK117-minC (labeled green) and PB103:pIKE107-RFP-minC (labeled red). The culture was exposed to a 15 min, 0.25 nM anhydrotetracycline (aTc) pulse and after 2 h, the culture was exposed to a 30 min, 10 mM IPTG pulse. The culture was effectively grown for 90 min post-IPTG exposure and 4 h post-aTc exposure and imaged using TRITC and FITC filters. Scale bar is 50 µm.

Although PB103:pIKE107-RFP-minC cells displayed uniform elongation upon continuous exposure to 1 nM anhydrotetracycline, the response time (i.e. the time taken for RFP and MinC expression) was much longer than that observed upon continuous exposure of PB103:pTAK117-minC cells to 2 mM IPTG. For instance, the anhydrotetracycline-induced population reached maximal cell lengths after 4 h, while their IPTG-induced counterparts required only 2.5 h to attain this stage (Figure 5). Our observation that both the fluorescence and the MinC-induced cell elongation is delayed in PB103:pIKE107-RFP-minC compared to PB103:pTAK117-minC may be due to the higher transcription efficiency of the pTrc-2 promoter relative to pLtetO-1 and the higher repression efficiency of cI compared to the TetR [2]. Our studies also revealed that just a 15 min exposure of PB103:pIKE107-RFP-minC cells to 0.5 nM anhydrotetracycline was sufficient to trigger cell elongation. After 210 min of culture in LB medium (50 mg/L ampicillin, no anhydrotetracycline), 55% of the aTc nanorecorder cell population was in class 1 (0.5 µm–4 µm), 9% in class 2 (4.1 µm–8 µm) and 36% of the population in class 3 (8.1 µm–16 µm). These results demonstrate the pulsed exposure also works with a anhydrotetracycline-inducible system. Independent experiments wherein a mixed culture of PB103:pTAK117-minC and PB103:pIKE107-RFP-minC cells were exposed sequentially to IPTG and anhydrotetracycline demonstrated the utility of these nanorecorders to simultaneously respond and generate a time-dependent response to two different chemical stimuli (Figure 6).

### Summary

We have shown that the facile modification of an existing genetic toggle switch can be exploited to generate a robust, biologically-based “nanorecorder” that could potentially be adapted to detect, recall and record a wide range of chemical stimuli. By embedding the expression of MinC, a cell division inhibitory protein, within a standard toggle switch framework, we demonstrated that a transient exposure to a selected signal molecule (namely IPTG or anhydrotetracycline) was sufficient to trigger a long-term cell elongation response in more than one-third of the exposed cells. As shown here, this induced response can be used to predict the time elapsed since signal exposure, thereby providing a time stamp to the exposure event. Also, slow growing bacterial cells harboring this gene circuit would be capable of recording longer time intervals post-signal exposure. While these biological nanorecorders exhibit many of the desired features that were designed into them, they are relatively imperfect devices, at least in terms of their sensitivity (in the case of IPTG, requiring millimolar instead of micromolar or nanomolar concentrations of the signal molecules), their fidelity (a 33% response rate instead of 100%) and required exposure times (minutes instead of seconds). Despite this, we believe these results provide the first instance of a synbio-inspired approach to use organisms as event-recording devices. We are currently looking at expanding the scope of this work to construct real-world nanorecorders for sensing pollutants and toxins (such as heavy metals, phenols) and clinically useful metabolites (nitrates, urea).

## Supporting Information

Figure S1 **Schematic restriction maps of A) pTAK117-minC and B) pIKE107-RFP-minC.**
(TIF)

Figure S2 **DNA sequence of the synthesized** ***Sph*** **I-** ***Kpn*** **I fragment.** RBS denotes the Ribosome Binding Site, while −10 and −35 represent the consensus −35 and −10 sequences of the pTrc2 promoter.
(TIF)

Figure S3 **A. Fluorescence decay of GFP following short-term IPTG exposure.** BL21(DE3) cells transformed with pET15b-GFP were grown at 37°C in LB medium supplemented with 50 mg/L ampicillin to mid-log phase (OD_600 nm_∼0.5) and were subcultured at a 1∶ 30 dilution into LB medium supplemented with 50 mg/L ampicillin and 1 mM IPTG. Following subculture, 1 ml aliquots were withdrawn at set time intervals (15 min, 30 min, 45 min and 60 min), briefly spun to remove IPTG and the pelleted cells were resuspended and cultured as before in 1 ml IPTG-less LBamp50 medium. The cells were observed under a fluorescence microscope at hourly intervals for a total of 10 h post-transfer to IPTG-less LBamp50 medium. Images were adjusted for adjusted for background intensities before recording cellular fluorescence intensities. Image processing was done using the software MetaMorph Basic (Molecular Devices Inc. version 7.7) while average fluorescence intensities and standard deviation were calculated using MS Excel. **B. Schematic restriction map of pET15b-GFP.** The coding sequence of GFP was amplified from pGFP [4] using primers having *Nde*I and *BamH*I overhangs and cloned into *Nde*I-*BamH*I digested pET15b (Novagen Inc. USA).
(TIF)

Figure S4 **Relative efficacy of cell division inhibition.** Visible light images of PB103 cells expressing MinC under A) uninduced and B) induced conditions and SfiA under C) uninduced and D) induced conditions. MinC expression was initiated by transferring freshly grown culture (OD_600 nm_∼0.3) of PB103:pDR175 (grown at 31°C in LB medium supplemented with 25 mg/L spectinomycin) to 42°C. SfiA expression was elicited by addition of 2 mM IPTG to a freshly grown culture (OD_600 nm_∼0.3) of PB103:λ DR144 (grown at 37°C in LB medium supplemented with 50 mg/L ampicillin). Cells were observed following 2 h 30 min of induction. Scale bar is 20 µm.
(TIF)

Figure S5 **Response of PB103:pTAK117 cells to short-term IPTG exposure.** A freshly grown culture of PB103:pTAK117 was used to inoculate LBamp50 supplemented with 2 mM IPTG and 10 mM IPTG followed by culturing the cells for 30 min at 31 deg C at 225 rpm. After 30 min, cells were washed off IPTG, cultured in LB amp50 only and visualized after 3 hours. A, B are visible and UV-light images, respectively, of cells exposed to 2 mM IPTG for 30 minutes while C,D are visible and UV-light images respectively, of cells exposed to 10 mM IPTG for 30 minutes. E and F represent visible and UV-light images respectively, of the control treatment wherein cells were grown in continuous presence of LBamp50 supplemented with 2 mM IPTG. Scale bar is 20 µm.
(TIF)

Text S1 **Optimization of the MinC reporter system.**
(DOC)
