# Supplementary figures and images for: The Bacterial Nanorecorder: Engineering E. coli to Function as a Chemical Recording Device
Source: PLoS One. 2011 Nov 23;6(11):e27559. doi: 10.1371/journal.pone.0027559 (PMC3223186; doi:10.1371/journal.pone.0027559)

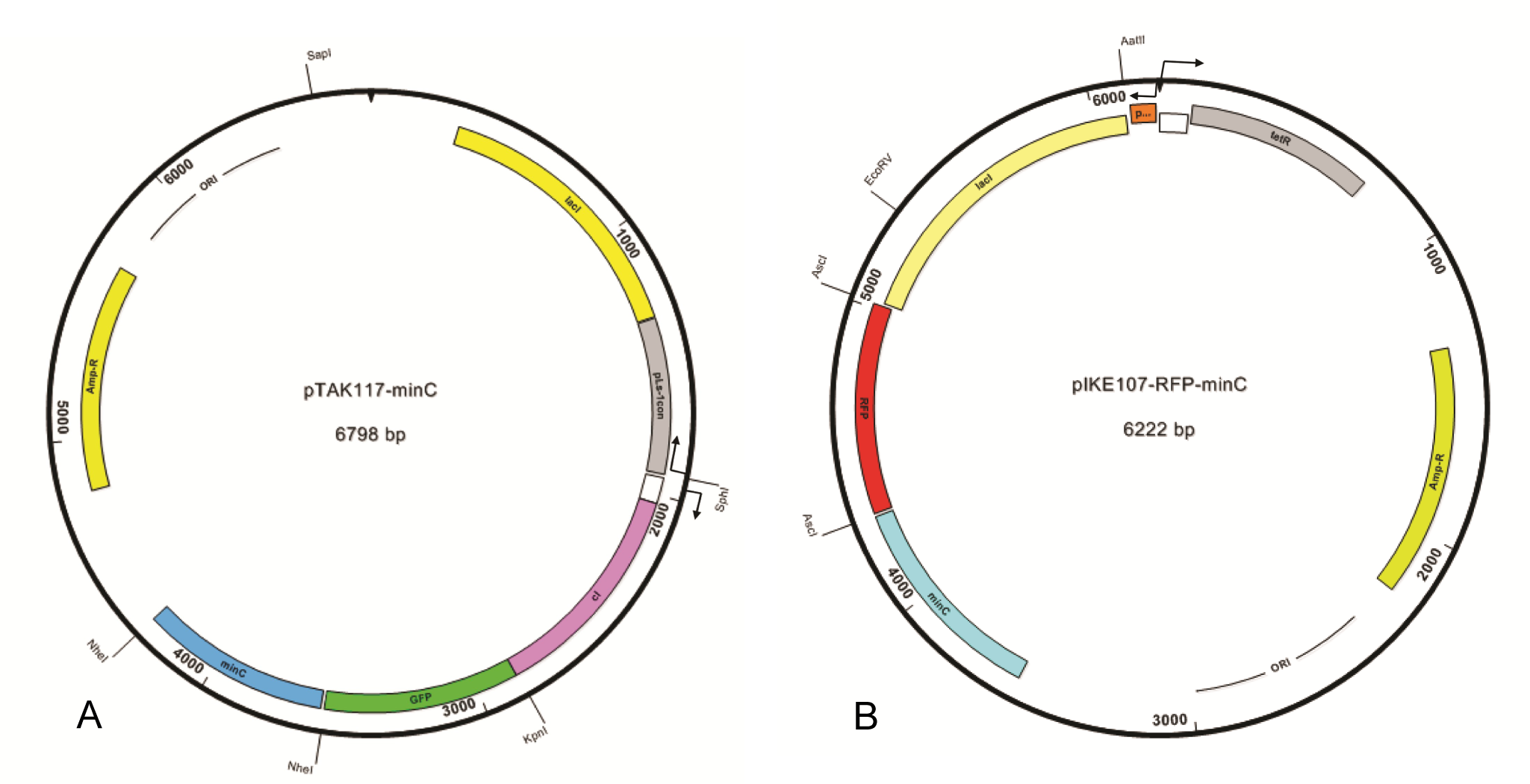

Supplement: Figure S1 — Schematic restriction maps of A) pTAK117-minC and B) pIKE107-RFP-minC. (TIF) [file pone.0027559.s001.tif]

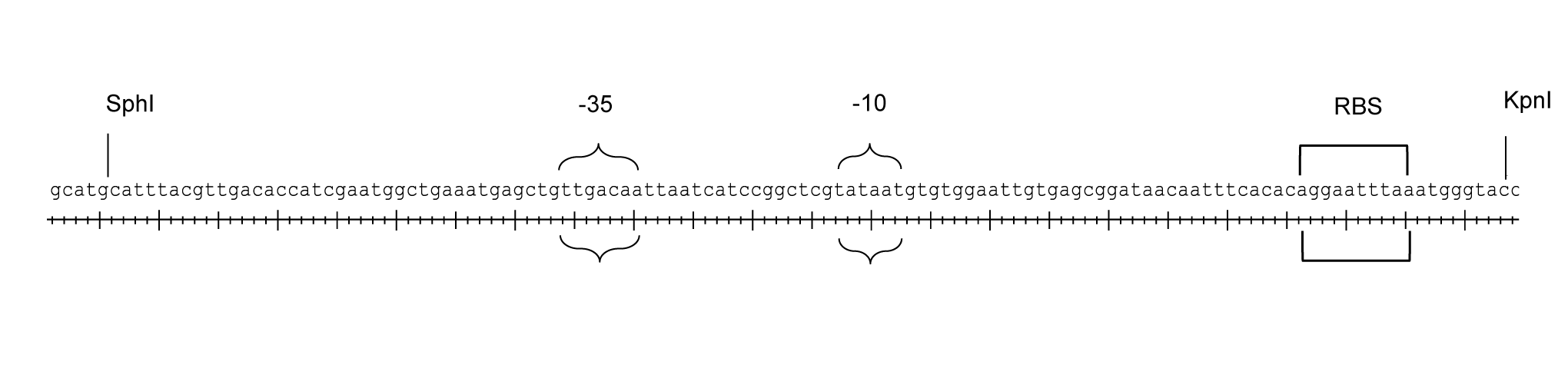

Supplement: Figure S2 — DNA sequence of the synthesized Sph I- Kpn I fragment. RBS denotes the Ribosome Binding Site, while −10 and −35 represent the consensus −35 and −10 sequences of the pTrc2 promoter. (TIF) [file pone.0027559.s002.tif]

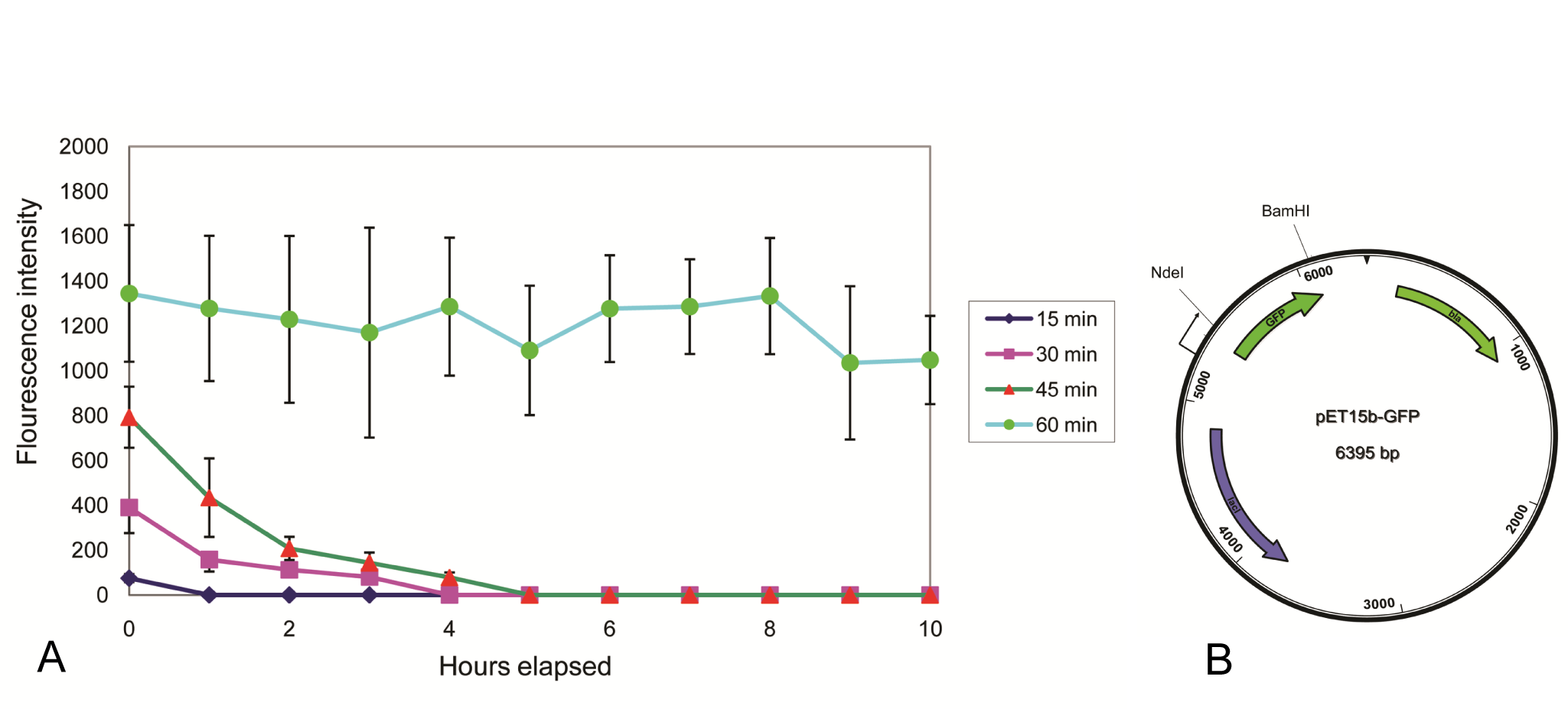

Supplement: Figure S3 — A. Fluorescence decay of GFP following short-term IPTG exposure. BL21(DE3) cells transformed with pET15b-GFP were grown at 37°C in LB medium supplemented with 50 mg/L ampicillin to mid-log phase (OD600 nm∼0.5) and were subcultured at a 1∶ 30 dilution into LB medium supplemented with 50 mg/L ampicillin and 1 mM IPTG. Following subculture, 1 ml aliquots were withdrawn at set time intervals (15 min, 30 min, 45 min and 60 min), briefly spun to remove IPTG and the pelleted cells were resuspended and cultured as before in 1 ml IPTG-less LBamp50 medium. The cells were observed under a fluorescence microscope at hourly intervals for a total of 10 h post-transfer to IPTG-less LBamp50 medium. Images were adjusted for adjusted for background intensities before recording cellular fluorescence intensities. Image processing was done using the software MetaMorph Basic (Molecular Devices Inc. version 7.7) while average fluorescence intensities and standard deviation were calculated using MS Excel. B. Schematic restriction map of pET15b-GFP. The coding sequence of GFP was amplified from pGFP [4] using primers having NdeI and BamHI overhangs and cloned into NdeI-BamHI digested pET15b (Novagen Inc. USA). (TIF) [file pone.0027559.s003.tif]

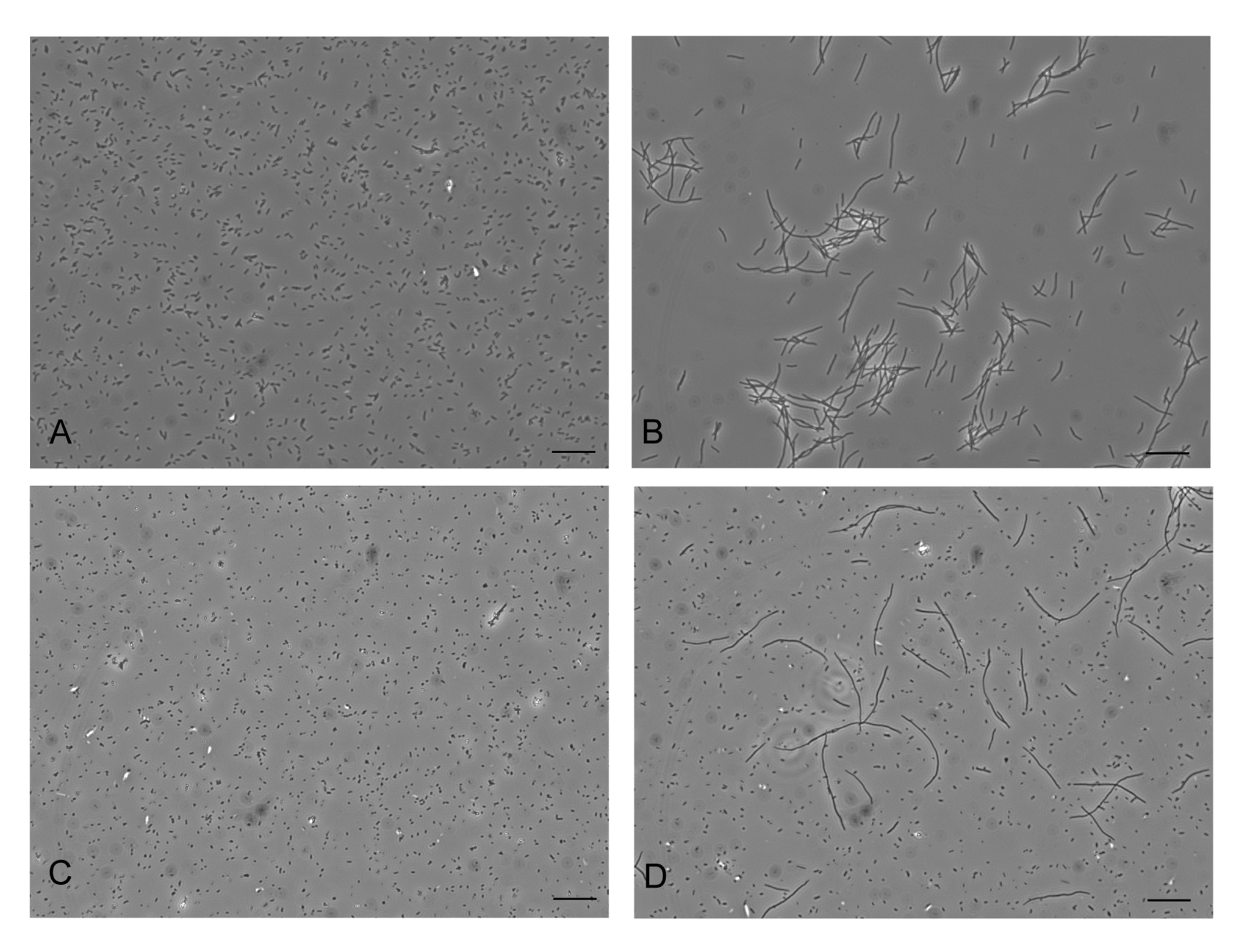

Supplement: Figure S4 — Relative efficacy of cell division inhibition. Visible light images of PB103 cells expressing MinC under A) uninduced and B) induced conditions and SfiA under C) uninduced and D) induced conditions. MinC expression was initiated by transferring freshly grown culture (OD600 nm∼0.3) of PB103:pDR175 (grown at 31°C in LB medium supplemented with 25 mg/L spectinomycin) to 42°C. SfiA expression was elicited by addition of 2 mM IPTG to a freshly grown culture (OD600 nm∼0.3) of PB103:λ DR144 (grown at 37°C in LB medium supplemented with 50 mg/L ampicillin). Cells were observed following 2 h 30 min of induction. Scale bar is 20 µm. (TIF) [file pone.0027559.s004.tif]

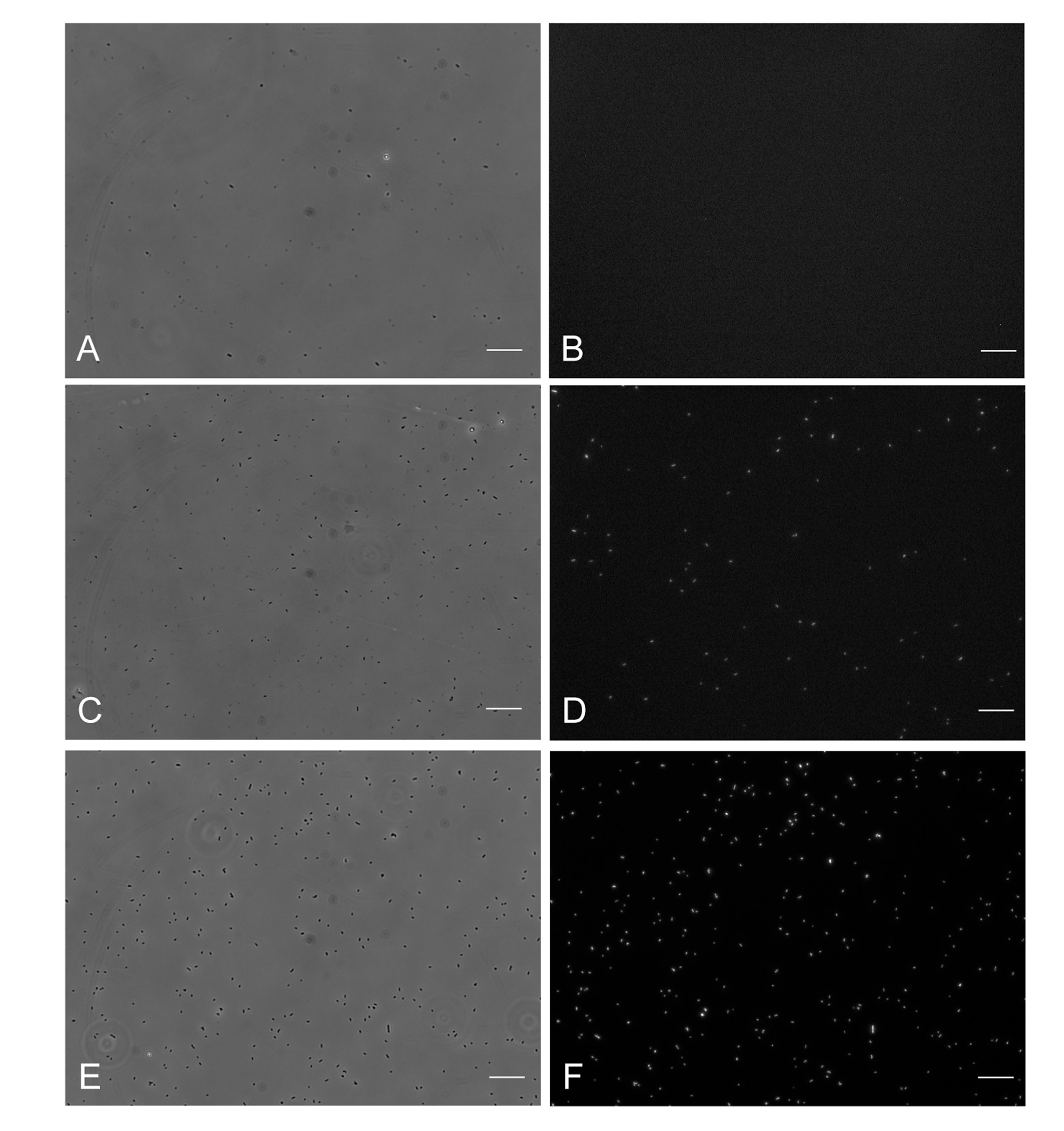

Supplement: Figure S5 — Response of PB103:pTAK117 cells to short-term IPTG exposure. A freshly grown culture of PB103:pTAK117 was used to inoculate LBamp50 supplemented with 2 mM IPTG and 10 mM IPTG followed by culturing the cells for 30 min at 31 deg C at 225 rpm. After 30 min, cells were washed off IPTG, cultured in LB amp50 only and visualized after 3 hours. A, B are visible and UV-light images, respectively, of cells exposed to 2 mM IPTG for 30 minutes while C,D are visible and UV-light images respectively, of cells exposed to 10 mM IPTG for 30 minutes. E and F represent visible and UV-light images respectively, of the control treatment wherein cells were grown in continuous presence of LBamp50 supplemented with 2 mM IPTG. Scale bar is 20 µm. (TIF) [file pone.0027559.s005.tif]
