# Supplementary material for: The Bacterial Nanorecorder: Engineering E. coli to Function as a Chemical Recording Device
Source: PLoS One. 2011 Nov 23;6(11):e27559. doi: 10.1371/journal.pone.0027559 (PMC3223186; doi:10.1371/journal.pone.0027559)
Supplement: Text S1 — Optimization of the MinC reporter system. (DOC) [file pone.0027559.s006.doc]

***Optimization of the MinC reporter system***

*minC as a reporter gene*

Prior to engineering the nanorecorder’s genetic circuit, we evaluated four different *E. coli* host cell strains (DH5α, GM2929, CSH50 and PB103) for their endogenous cell lengths. Of these, only PB103 host cells displayed a cell population with minimal variation in cellular lengths (data not shown). Accordingly, PB103 was chosen as the bacterial “chassis” for the bacterial nanorecorder. In order to devise an effective bacterial time-stamp, we decided to couple a chemically sensitive toggle switch to a cell division inhibition signal and a chemically specific fluorescence signal. This co-expression concomitantly results in cell elongation along with a sustained chemo-specific fluorescence signal (red or green, depending on the chemical inducer). The length of the elongating bacterial cell is used as the parameter to calculate the time elapsed following chemical exposure.

In *E. coli*, the formation of two equal-sized daughter cells is initiated by the formation of the Z-ring (composed of polymerized FtsZ filaments) at the mid-cell. Both SfiA [1] and MinC [2] inhibit cell division by interfering with FtsZ polymerization. It has also been shown that independent over-expression of SfiA [3] and MinC [4] cause a global block in cell division, resulting in the formation of long non-septate filaments, a phenomenon known as filamentation*.* In order to develop the bacterial version of a time-stamp, we compared the relative efficacy of SfiA and MinC overexpression in triggering uniform filamentation in wild-type PB103 cells. Overexpression of MinC and SfiA in PB103 cells resulted in the desired phenotype (elongated cells), but with differing population variability. Overexpression of MinC (Figure S4-B) resulted in a uniformly elongated cell population, while the cell population overexpressing SfiA (Figure S4-D) displayed considerable variability in the cell lengths, implying incomplete or inefficient inhibition of cell division. Both SfiA and MinC block cell septation by interfering with FtsZ polymerization. However, SfiA functions by obstructing FtsZ’s GTPase activity [5] while MinC compromises the ability of FtsZ filaments to be in a solid-like gel state thereby preventing lateral interactions between FtsZ filaments [6]. This may explain why MinC overproduction resulted in better inhibition of cell division. We therefore chose to couple MinC expression to the toggle switch to obtain a filamentation phenotype in PB103 cells.

*Achieving physiologically relevant concentrations of MinC*

Gardner et al.[7] noted that although all genes and promoters that comprised the toggle switch were placed on a single plasmid, they could, in principle, be divided into two separate plasmids without altering functionality of the switch. Our experiments, based on this premise, however, did not yield the expected results. PB103 host cells co-transformed with pTAK117 and pTrc-minC showed filamentation both in the presence or absence of IPTG. Furthermore, this background filamentation was independent of the strength of the RBS sequence used for controlling MinC translation. This pronounced background cell elongation was indicative of “leaky” MinC expression, likely due to titration of the lacI repressor protein. This suggested that cloning the *minC* insert directly on pTAK117 might be required to prevent leaky MinC expression. Accordingly, the *minC* insert (with different RBSs) was cloned non-directionally into *Nhe*I-digested pTAK117, transformed into XL1-Blue host cells and the recombinants with *minC* in the sense orientation were identified using a simple phenotypic screen.

Briefly, plasmid DNA was isolated from putative recombinant clones (irrespective of the orientation of the cloned *minC* coding sequence) and independently transformed into PB103 host cells. A single colony from each PB103 clone was cultured in liquid LBamp50 + IPTG (LB media supplemented with 50 mg/L ampicillin and 2 mM IPTG) and observed microscopically after allowing 2 h for cell elongation. We hypothesized that cell elongation under these conditions would be possible only in those recombinants wherein the *minC* coding sequence was ligated in the sense orientation. Sequencing of plasmid DNA isolated from those clones exhibiting cell elongation in the presence of IPTG confirmed that the *minC* coding sequence was ligated in the sense orientation. Of the three ribosome-binding sequences, only RBS^C^ (a weak RBS) resulted in minimal background cell elongation (i.e. negligible cell elongation in the absence of IPTG).

At normal levels of expression, both MinC and MinD cooperate to form an inhibitor complex to non-specifically block cell division at all potential sites in *E. coli*, but is countered by MinE at the mid-cell, thereby ensuring proper placement of the division septum in *E. coli* [4]. Therefore, balanced expression of MinCD and MinE are necessary to maintain the normal division pattern. deBoer et al. [4] also showed that MinC overexpression inhibits cell division in the absence of MinD and is not suppressed by normal levels of MinE. Our goal in designing a bacterial time-stamp device was to manipulate expression levels of the least number of gene products to obtain the desired phenotypic response - namely cell filamentation. Thus, controlling MinC levels seemed to be the most attractive option to achieve inducible cell filamentation.

However, the extent of MinC-mediated cell division inhibition is also dependent on the precise intra-cellular MinC concentrations. For instance, deBoer et al. [4] showed that the accumulation of a large number of filamentous cells was possible only when *minC* was expressed on a high-copy plasmid. Indeed, MinC production from a single-copy or a moderate copy plasmid was unable to perturb cell division. When the wild-type *minC* RBS on the moderate copy plasmid was replaced with a stronger RBS, the resultant increase in MinC concentration was sufficient to induce cell filamentation. As suggested by deBoer’s study, our results confirmed that optimizing the strength of the upstream RBS and fine-tuning *in vivo* MinC levels by expressing MinC *in cis* (instead of *in trans*) was critical to support MinC-dependent division inhibition as a reporter phenotype.

**References**

1. Gottesman S, Halpern E, Trisler P (1981) Role of sulA and sulB in filamentation by lon mutants of Escherichia coli K-12. J Bacteriol 148(1): 265-273.

2. Raskin DM, de Boer PA (1999) MinDE-dependent pole-to-pole oscillation of division inhibitor MinC in Escherichia coli. J Bacteriol 181(20): 6419-6424.

3. Bi E, Lutkenhaus J (1993) Cell division inhibitors SulA and MinCD prevent formation of the FtsZ ring. J Bacteriol 175(4): 1118-1125.

4. de Boer PA, Crossley RE, Rothfield LI (1989) A division inhibitor and a topological specificity factor coded for by the minicell locus determine proper placement of the division septum in E. coli. Cell 56(4): 641-649.

5. Dajkovic A, Mukherjee A, Lutkenhaus J (2008) Investigation of regulation of FtsZ assembly by SulA and development of a model for FtsZ polymerization. J Bacteriol 190(7): 2513-2526.

6. Dajkovic A, Lan G, Sun SX, Wirtz D, Lutkenhaus J (2008) MinC spatially controls bacterial cytokinesis by antagonizing the scaffolding function of FtsZ. Curr Biol 18(4): 235-244.

7. Gardner TS, Cantor CR, Collins JJ (2000) Construction of a genetic toggle switch in *Escherichia coli*. Nature 403(6767): 339-342.
